# A comparative study of early functional outcomes in undisplaced neck of femur fracture treated with partially threaded and fully threaded cannulated screw fixation in patients above 60 years age

**DOI:** 10.12669/pjms.39.5.7330

**Published:** 2023

**Authors:** Muhammad Zarak Awais, Eman Salik, Syed Hassan Raza Bokhari, Muhammad Tahir Iqbal

**Affiliations:** 1 Muhammad Zarak Awais, MBBS Department of Orthopedic Surgery, Mayo Hospital, Lahore, Pakistan; 2 Eman Salik, MBBS. Department of Orthopedic Surgery, Mayo Hospital, Lahore, Pakistan; 3 Syed Hassan Raza Bokhari, FCPS (Orthopedic Surgery) Punjab Rangers Teaching Hospital, Lahore, Pakistan; 4 Muhammad Tahir Iqbal, MBBS. Department of Orthopedic Surgery, Mayo Hospital, Lahore, Pakistan

**Keywords:** Cannulated screw, Functional outcome, RUSH score, Hip Harris score

## Abstract

**Objective:**

To compare the partially threaded versus fully threaded cannulated screw fixation methods in stable neck of femur fracture in terms of early functional outcomes in patients of age 60 and above.

**Methods:**

A randomized controlled trial was conducted at Orthopedic Unit-II, Mayo Hospital Lahore, Pakistan from July 2021 to July 2022. A total of 82 (41 in each group) patients of both genders, aged 60 or above were included. All patients had garden Type-I or II and were mobile before they fractured the femoral neck were included. In Group-A, cannulated screw fixation (cancellous screws 6 mm) was done using partially threaded screws while fully threaded screws (cancellous screws 6 mm) were used in Group-B. Patients were followed up at six weeks, three months and six months interval for “Radiographic Union Scale for Hip (RUSH)” and “Harris Hip score”.

**Results:**

In a total of 82 (41 in each group) patients, 66 (80.5%) we male. At 6^th^ week (p=0540) and 3^rd^ month (p=0.653) postoperatively, no significant differences were seen between groups for Hip Harris score. However, at 6^th^ month, functional outcome of Group-B patients was significantly better as compared to Group-A (p=0.038). Mean RUSH score in Group-A and in Group-B at 6^th^ month postoperative was 25.45±2.73 and 30.52±2.39 (p<0.001).

**Conclusion:**

Fully threaded cannulated screw fixation is better in treating undisplaced neck of femur fracture as compared to partially threaded cannulated screw fixation in terms of early functional outcomes among the age group of 60 years and above.

***RCT Registration number:*** NCT05587660.

## INTRODUCTION

Fracture neck of femur is common in the elderly. An increase in cases of osteoporosis in elderly people is one of the major reasons for hip fracture.^1^ Mortality rates between 20-30% are reported due to the femur injury and the mortality rate in the elderly patients is expected to increase in the next 20 years.^2^ A large number of cases of hip fracture shake the healthcare system and the economy.3 Surgical intervention is only option for treating neck of femur fractures.^3^

There are several surgical fixing techniques for neck of femur fracture including bipolar hemiarthroplasty, AMP, THR and cannulated screw fixation. Bipolar hemiarthroplasty is generally preferred choice in treating unstable neck of femur fracture. This method was introduced to avoid the risk of endoprosthesis arthritis.^4^ However several studies have reported an increase in dislocation rate after Bipolar hemiarthroplasty.^5^,^6^

The most popular method in undisplaced neck of femur fracture include internal fixation by cannulated screws in which screws can be inserted with minimal invasion and X-ray guidance.. Partially or fully threaded Screws can be used however studies on fully threaded screw fixation method are inconclusive.^7^ If screws insertion is not performed in a proper way, they could cut through the upper and lower cortices.^8^ A study analyzing prognosis of variable pitch fully threated screws for fixation of FNF in comparison to partially threaded screws revealed no statistically significant differences in terms of non-union (8.7% vs. 0%, p= 0.159).^9^

Sometimes intracapsular fracture and synovial fluid, the blood supply to the femur head intervene in the healing process and causes severe and unpredictable complications. From all the above-mentioned literature, this study aimed to conduct for examining undisplaced neck of femur fracture among the age group of 60 and above to select the best treatment for them. The objective was to compare the partially threaded versus fully threaded cannulated screw fixation methods in stable neck of femur fracture in terms of early functional outcomes in patients of age 60 and above. Our hypothesis was that fully threaded cannulated screw fixation is better in treating undisplaced neck of femur fracture than partially threaded cannulated screw fixation in terms of early functional outcomes among the age group of 60 and above.

## METHODS

This randomized controlled trial was conducted at The Orthopaedic Unit-II, Mayo Hospital, Lahore Pakistan from January 2022 to July 2022. This trial was registered at clinicaltrials.gov as NCT05587660. Sample size of 82 patients (41 in each group) was calculated considering 95% confidence level, 80% power of test with expected percentage non-union with fully threaded screws as 8.7% and 0% with partially threaded screws.^9^ Non probability convenient sampling technique was utilized.

### Inclusion criteria:

Patients of both genders, aged 60 or above. All these patients had garden Type-I or II and were mobile before FNF. Intra capsular fracture was described as a fracture of a bone that usually located inside a joint capsule.

### Exclusion criteria:

Patients having open fracture or those with present or past history of chronic kidney disorder, hemorrhage, nervous stroke or unwilling to be part of this study. Approval from “Institutional Ethics Committee” was acquired (Letter number: 227/RC/KEMU, Dated: March 15^th^ 2021). Informed and written consents were obtained. Patients were admitted through outpatient department (OPD) or emergency department after taking complete history, physical examination and randomly allocated to study groups (A and B). Patients were asked to follow up at six weeks, three months and six months interval.

In both groups fixation was done by reverse triangle technique. In Group-A, cannulated screw fixation (cancellous screws 6mm) was done using partially threaded screws. In this procedure, first step was insertion of three guide wires under image. The 1^st^ guide wire was inserted immediately above calcar and into femoral head. The second wire was placed posteriorly adjacent to posterior cortex of neck on lateral view. Finally, third guide wire was placed anteriorly and superiorly. Drilling of lateral cortex was done before insertion of screws. Then partially threaded screw fixation was done before removal of guide wires using washers. In group B patients the procedure was same as in Group A, however, instead of partially threaded screws, fully threaded screws (cancellous screws 6mm) were used.

Patients were kept at touch-down weight bearing for duration of 10-12 weeks. Older patients were allowed protected weight bearing with a walker. Patients who were not safely ambulate were encouraged to mobilize to a chair to minimize pulmonary complications. We conducted plain radiographs to examine the position of pelvis, hip, knee, and femur in anteroposterior and lateral views. The Harris Hip Score (HHS Score) was used to evaluate fracture healing. RUSH Score was calculated with “Rradiographic Union Score for Hip (RUSH)”, designed to improve intra-and inter-observer reliability when describing the radiographic healing of FNF.

Data was collected from patients of both groups A and B according to Harris Hip score. Quantitative variables like age, RUSH Score and Harris Hip Score was presented as mean and standard deviations. Qualitative variables like gender, Radiological outcomes and functional outcomes were presented as frequency and percentage. Comparison between fully threaded screen and partially threaded screen was done by applying chi-square test. Quantitative data like age was compared using independent sample t-test. P<0.05 was considered as significant.

### Statistical Analysis:

Data was entered and analyzed by “Statistical Package for Social Sciences (SPSS)” version 26.0.

## RESULTS

In a total of 82 patients (41 in each group), 66 (80.5%) were males and 16 (19.5%) females representing a male to female ratio of 4.1:1. Mean age of patients in Group-A and in Group-B was 68.92±6.28 and 69.53±5.88 years (p=0.651). Age ranged between 60-80 years in both study groups. Comparisons of gender and age of the patients in both study groups is shown in Table-I.

**Table-I:** Comparison of Gender and Age of patients in both Study Groups (N=82)

Characteristics	Group-A (n=41)	Group-B (n=41)	P-Value
Gender			0.094
Male	30(73.17%)	36(87.80%)	
Female	11(26.83%)	5(12.20%)	
Age (years)	68.92±6.28	69.53±5.88	0.651

At 6^th^ week (p=0.540) and three months (p=0.653) postoperatively, no significant difference was seen between groups for HHS score. At 6^th^ month Group-B had significantly better outcomes in terms of HSS score as compared to Group-A patients (p-value=0.038). Comparison of HHS scores in between both study groups is shown in Table-II.

**Table-II:** Harris Hip Score in Treatment Groups at 6^th^ Week, three-month and six-month Post Treatment (N=82).

Post Treatment HHS		Group-A (n=41)	Group-B (n=41)	P-Value
6^th^ Week	Poor	17(41.46%)	16(39.02%)	0.540
	Fair	21(51.22%)	24(58.54%)	
	Good	3(7.32%)	1(2.44%)	
	Excellent	0(0%)	0(0%)	
3^rd^ Months	Poor	11(26.83%)	10(24.39%)	0.653
	Fair	15(36.59%)	19(46.34%)	
	Good	15(36.59%)	12(29.27%)	
	Excellent	0(0%)	0(0%)	
6^th^ Months	Poor	7(17.07%)	1(2.44%)	0.038
	Fair	9(21.95%)	5(12.20%)	
	Good	17(41.46%)	19(46.34%)	
	Excellent	8(19.51%)	16(39.02%)	

Poor<70; Fair=70-80; Good=80-90; Excellent=90-100

Mean RUSH score in Group-A, and in Group-B at 6^th^ week postoperative was 15.46±1.83 and 16.16±2.82 (p=0.186). Mean RUSH score in Group-A and in Group-B at 3^rd^ month postoperative was 19.18±2.37 and 23.68±2.57, and at this point, significant difference was seen for Group-B patients in terms of RUSH score (p<0.001). Mean RUSH score in Group-A and in Group-B at 6^th^ month postoperative was 25.45±2.73 and 30.52±2.39 (p<0.001).

## DISCUSSION

Results of this study showed that at 6^th^ week and 3^rd^ month postoperative no significant difference was seen between groups for Hip Harris score. However, at 6^th^ month functional outcome of fully threaded screws (FTS) was significantly better when compared to partially threaded screws (PTS). (p=0.038). For RUSH score no significant difference was seen for RUSH score between groups. However, at 3^rd^ (Group-A: 19.18 vs. Group-B: 23.68, p-value<0.001) and 6^th^ month (Group-A: 25.45 vs. Group-B: 30.52, p<0.001) significant difference was seen for RUSH score between groups.

Patients in Group-B had higher RUSH score as compared to Group-A. Schaefer et al^10^ revealed that replacing the posterior PTS with an FTS in FNF with posterior comminution resulted in beneficial outcomes in comparison to three PTS fixation models. Zhang and colleagues shared that utilization of triangle configuration of PTS with two headless FTSs resulted in improvement in the clinical and biochemical outcomes of unstable FNF in comparison to PTS alone.^11^ Weil et al.^12^ found that significant reduction in femoral neck shortening rates following cannulated screw fixation when FTSs of inverted triangular configuration were employed in comparison to PTSs. Contemporary data has also shown that use of FTS increased time to union and nonunion rates.^13^ These findings are in line with the result of study as we used RUSH score for radiological union scale for hip showed that significantly higher RUSH score was observed in Group-B (FTS) patients.^14^ Meanwhile, others have found that, in comparison with fully threaded headless cannulated screws (FTHCS), PTS provided a significantly shorter union time and lower rates of complications (9% vs. 36%).^13^ Chiang and coworkers found that FTHCS were unable to avoid femoral neck shortening as well as varus collapse following fracture fixation.5 They also showed that relatively similar complication rates between both approaches (18% vs. 21%).^5^ Another study analyzing prognosis of variable pitch FTHCS for fixation of FNF in comparison to PTS revealed no statistically significant differences in terms of non-union (8.7% vs. 0%, p= 0.159), avascular necrosis rates (17.4% vs. 23.5%, p=0.744) and good and excellent rates of Harris scores (73.9% vs. 82.4%, p=0.443).^9^ The proximal fracture fragment and PTS may move lateraldistally, resulting in neck shortening and lateral screw protrusion, especially in comminuted fractures.^14^

Some other researchers have demonstrated that FTS are more biomechanically stable especially among cases of vertical fracture models.^14^ The new configurations may provide a useful solution providing combination of the advantages of these two kinds of screws but we need more comparative studies to further establish the findings of what is already known about the fully threaded screws and other configuration of screws.^15^-^17^ Walker et al found that three cannulated screws were relatively more stronger when compared to two screws but the difference did not turned out to be statistically different (p>0.05).^18^ The use of FTSs in the fixation of FNF is still debatable and needs more research especially regarding ‘when’ and ‘how’ to use these screws. There is also a debate about which screws are more preferable, or how many screws must be used, and which screws are biomechanically better in different bone models.^18^

**Table-III:** RUSH Score in Treatment Groups at 6^th^ Week, 3^rd^ Month and 6^th^ Month Post Treatment.

RUSH Score	Group-A (n=41)	Group-B (n=41)	P-Value
6^th^ Week	15.46±1.83	16.16±2.82	0.186
3^rd^ Month	19.18±2.37	23.28±2.57	<0.001
6^th^ Month	25.45±2.73	30.52±2.39	<0.001

### Limitations:

It includes a single center study with a relatively modest sample size. The outcome was noted at maximum follow up interval of six months, so future studies employing longer duration of follow ups can certainly add to what has been found so far.

## CONCLUSION

Results of this study demonstrate that fully threaded cannulated screw fixation is better in treating undisplaced neck of femur fracture as compared to partially threaded cannulated screw fixation in terms of early functional outcomes among the age group of 60 and above.

### Authors’ Contribution:

**MZA:** Idea, data collection, responsible for integrity of the study. **ES:** Data collection, drafting.

**SHRB:** Proof reading, corrections.

**MTI:** Literature review, discussion.
